# Miniaturized Versus Standard-Tract Percutaneous Nephrolithotomy for Renal Calculi Larger Than 2 cm: Tract Caliber Determines Hemorrhagic Morbidity Without Compromising Stone Clearance

**DOI:** 10.7759/cureus.116347

**Published:** 2026-09-16

**Authors:** Sajad A Malik, Omar Mukhtar, Yawar Nazir, Arif Hamid, Abdul Rouf Khawaja, Sajad Para, Saqib Mehdi, Syed Aadil S Andrabi, Shabbah Alam

**Affiliations:** 1 Department of Urology, Sher-i-Kashmir Institute of Medical Sciences, Srinagar, IND

**Keywords:** hemorrhage, kidney calculi, length of stay, mini-pcnl, percutaneous nephrolithotomy, stone-free rate, tract dilatation

## Abstract

Background

Percutaneous nephrolithotomy (PCNL) is the treatment of choice for renal calculi larger than 2 cm, but much of its morbidity arises from the access tract rather than from stone removal itself. Whether tract miniaturization preserves clearance at this stone burden, where a larger working channel might be presumed necessary, remains incompletely defined outside randomized settings. We compared perioperative outcomes of mini-PCNL and standard PCNL in a high-volume tertiary cohort.

Materials and methods

We retrospectively analyzed 215 consecutive adults undergoing PCNL for renal calculi larger than 2 cm between January 2022 and January 2026. Patients underwent mini-PCNL (16-20 Fr tract, 12-15 Fr nephroscope; n=100) or standard PCNL (24-30 Fr tract, 20-24 Fr nephroscope; n=115) according to surgeon preference. All procedures used prone, fluoroscopy-guided single-tract access with serial Amplatz dilatation and pneumatic lithotripsy. The primary outcome was the stone-free rate, defined as absence of residual fragments larger than 4 mm on computed tomography (CT) at four weeks. Secondary outcomes were operative time, hemoglobin decrement, hospital stay, and complications graded by the Clavien-Dindo classification. Differences are reported with 95% confidence intervals (CIs).

Results

Baseline characteristics were comparable. Stone-free rates did not differ (92% vs. 93.9%; absolute difference 1.9%; 95% CI -5.0 to 8.8; p=0.58), nor did operative time (80.9±18.2 vs. 76.4±21.3 minutes; mean difference -4.5; 95% CI -9.9 to 0.9; p=0.10) or overall complication rate (14% vs. 19.1%; p=0.31). Mini-PCNL produced a smaller hemoglobin decrement (0.95±0.56 vs. 1.48±0.71 g/dL; mean difference 0.53; 95% CI 0.36 to 0.70; p<0.001) and shorter hospital stay (2.4±0.8 vs. 3.1±1.1 days; mean difference 0.70; 95% CI 0.44 to 0.96; p<0.001).

Conclusions

At stone burdens above 2 cm, tract miniaturization reduced hemoglobin decrement by approximately one-third and shortened hospitalization by approximately 0.7 days, without loss of stone clearance or significant prolongation of operative time. Because puncture technique, dilatation method, lithotrite, and exit strategy were uniform between groups, tract caliber remains the principal variable distinguishing them. These findings support mini-PCNL as a reasonable default for large-volume non-staghorn calculi in experienced hands.

## Introduction

Urolithiasis is among the most common urological presentations worldwide, and its burden has risen over the past two decades with obesity, dietary transition, and ambient heat exposure [1]. Prevalence varies markedly across Asia, from approximately 1% to 19%, with South Asia among the higher-burden subregions [2]. The consequence for tertiary centers is a steady volume of large calculi presenting late and frequently already exceeding 2 cm.

Above this threshold, spontaneous passage is negligible, and both shockwave lithotripsy and ureteroscopy achieve substantially lower single-session clearance, so percutaneous nephrolithotomy (PCNL) is endorsed as the treatment of choice by both the European Association of Urology and the American Urological Association [3,4]. Its efficacy is not in dispute. Its morbidity, however, derives largely not from the removal of the stone but from the creation of the access required to reach it: in the Clinical Research Office of the Endourological Society Percutaneous Nephrolithotomy Global Study of 5,803 patients, hemorrhage was among the complications most consistently encountered after otherwise successful procedures [5].

This distinction identifies tract caliber, rather than the percutaneous approach itself, as the modifiable determinant of morbidity. Miniaturized PCNL, conventionally defined by tracts of 14-20 Fr, was developed on that premise, and pooled randomized data show reduced hemoglobin decrement and transfusion requirement with miniaturized access at the cost of modestly longer operative time [6]. The premise is least secure where it matters most, since as stone burden increases, the efficiency conferred by a larger working channel might reasonably offset its hemorrhagic penalty. We therefore compared stone clearance and perioperative morbidity between miniaturized and standard-tract PCNL in a consecutive tertiary cohort with renal calculi larger than 2 cm.

## Materials and methods

Study design and population

We retrospectively analyzed consecutive adults undergoing PCNL for renal calculi larger than 2 cm on non-contrast computed tomography (CT) at Sher-i-Kashmir Institute of Medical Sciences, a tertiary referral center in Srinagar, India, between January 2022 and January 2026. The institute's Institutional Ethics Committee approved the protocol (approval number: SIMS 127/IEC-SKIMS/2026-131) before data abstraction and waived individual consent given the retrospective design and anonymization of records. The study accorded with the Declaration of Helsinki. Exclusion criteria were uncorrected coagulopathy, untreated urinary tract infection, pregnancy, and complex staghorn calculi requiring multi-tract access. Of 215 eligible patients, 100 underwent mini-PCNL (16-20 Fr tract, 12-15 Fr nephroscope) and 115 standard PCNL (24-30 Fr tract, 20-24 Fr nephroscope). Allocation reflected surgeon preference; patients were not randomized.

Operative technique

All patients received antibiotic prophylaxis, and a sterile preoperative urine culture was a prerequisite. Under general anesthesia, a 5-6 Fr ureteric catheter was placed retrogradely at cystoscopy, and patients were turned prone. Retrograde pyelography opacified the collecting system, and the target calyx was punctured under fluoroscopic guidance with an 18-gauge needle, sited to permit clearance of the maximum stone burden through a single tract. The tract was then dilated with serial Amplatz dilators to the group-specific caliber, and a corresponding sheath was placed.

Stone fragmentation was performed using pneumatic lithotripsy in both groups. Fragments were retrieved with grasping forceps in the standard PCNL group, whereas in the mini-PCNL group, smaller fragments were additionally cleared by the hydrodynamic effect of pressurized irrigation through the sheath. Clearance was confirmed by nephroscopic inspection and on-table fluoroscopy. An antegrade double-J stent was placed in all patients, and a nephrostomy tube was sited at the surgeon's discretion. Only single-tract procedures were included.

Outcomes

The primary outcome was the stone-free rate, defined as absence of residual fragments larger than 4 mm on CT at four weeks. Secondary outcomes were operative time (from puncture to completion), hemoglobin decrement (the preoperative value minus the postoperative nadir), hospital stay, and complications graded using the Clavien-Dindo classification [7].

Statistical analysis

Continuous variables are presented as mean±standard deviation and were compared using the independent-samples (Student's) t-test with pooled variance (df=213). Categorical variables are presented as counts and percentages and were compared using the Pearson chi-squared test without continuity correction (df=1). Every reported p-value derives from one of these two tests, and the corresponding test statistic is tabulated alongside it; t was computed as mini-PCNL minus standard PCNL, so negative values denote lower values in the mini-PCNL arm. Absolute between-group differences are reported with 95% confidence intervals (CIs). To distinguish genuine absence of effect from inadequate power, standardized effect sizes were derived for every outcome, with 95% CI: Cohen's d, standardized to the pooled standard deviation, for continuous outcomes and risk ratios for categorical outcomes. Post hoc achieved power to detect the observed effect was computed for each comparison at a two-sided alpha of 0.05. A two-sided p<0.05 was considered significant. All analyses were performed using IBM SPSS Statistics for Windows, Version 22.0 (IBM Corp., Armonk, New York, United States).

## Results

Of the adults presenting with renal calculi larger than 2 cm during the study period, 215 met the protocol criteria: 100 undergoing mini-PCNL and 115 undergoing standard PCNL. All completed non-contrast CT at four weeks, so none was lost to outcome ascertainment (Figure 1).

**Figure 1:**
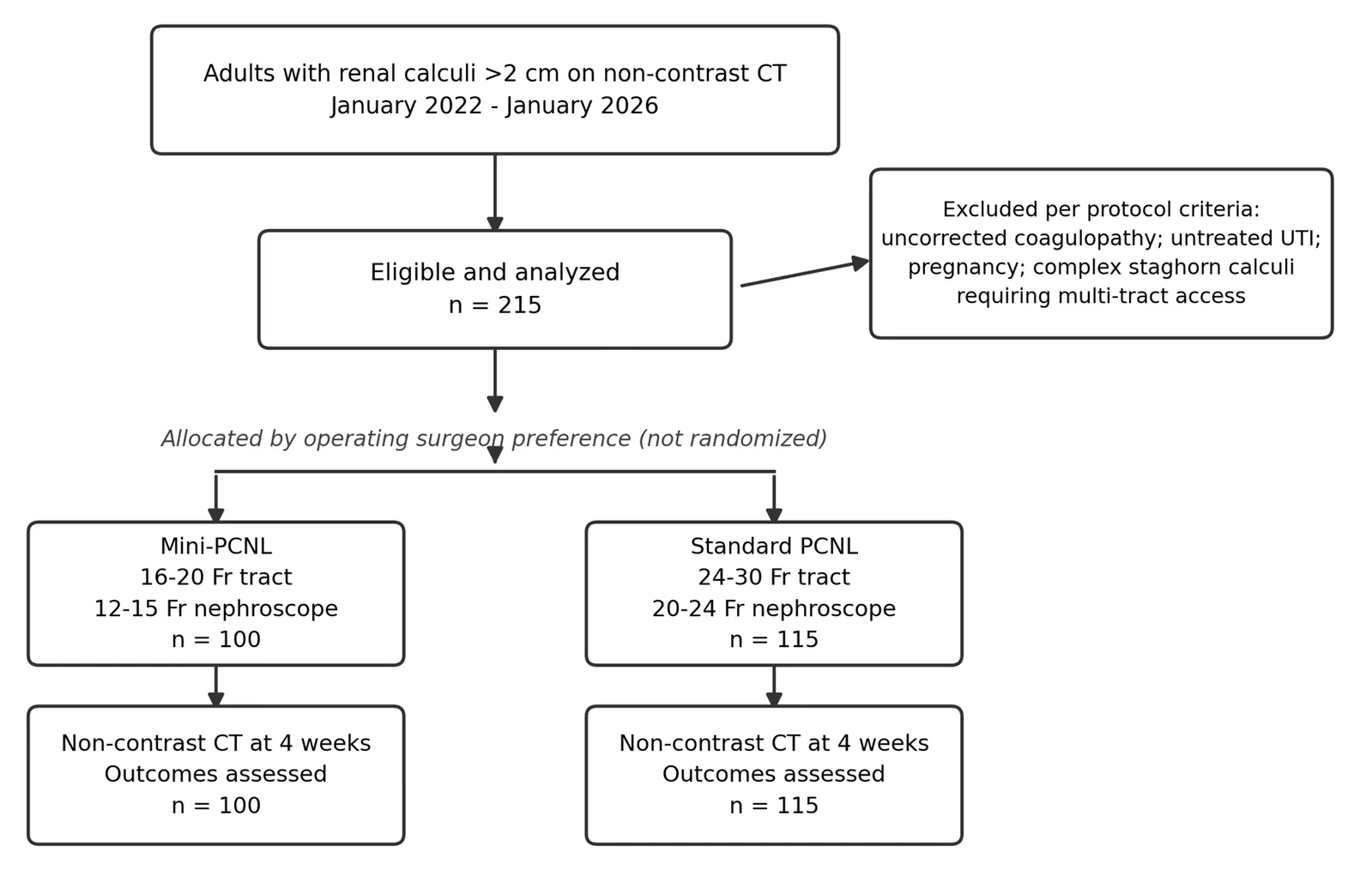
Derivation of the study cohort and allocation to treatment groups Allocation to tract caliber followed the operating surgeon's preference; patients were not randomized. CT: computed tomography; PCNL: percutaneous nephrolithotomy; UTI: urinary tract infection

The two groups were comparable at baseline in age, sex distribution, and laterality, and no variable differed significantly. The only appreciable separation was in stone size, marginally greater in the standard PCNL group. Although non-significant, the direction of this imbalance runs against the findings that follow, since stone size most influences both clearance and blood loss; any residual advantage would inflate rather than diminish the apparent performance of mini-PCNL, making the efficacy comparison conservative (Table 1).

**Table 1:** Baseline demographic and stone characteristics P-values are derived from the independent-samples t-test (continuous variables; df=213) or Pearson chi-squared test (categorical variables; df=1). PCNL: percutaneous nephrolithotomy

Characteristic	Mini-PCNL (n=100)	Standard PCNL (n=115)	Test statistic	P-value
Age (years), mean±SD	45.3±11.8	46.1±12.4	t=-0.48	0.63
Male, n (%)	70 (70)	78 (67.8)	χ²=0.12	0.73
Stone size (cm), mean±SD	2.6±0.4	2.7±0.5	t=-1.60	0.11
Right-sided stone, n (%)	58 (58)	63 (54.8)	χ²=0.23	0.64

Across the five study outcomes, the pattern was consistent. The two measures of perioperative cost, hemoglobin decrement and hospital stay, both favored mini-PCNL and reached significance, whereas stone clearance, operative time, and overall complication rate showed no significant separation. Every point estimate favored mini-PCNL except operative time, which was longer in that arm (Table 2).

**Table 2:** Perioperative and postoperative outcomes Negative t values indicate a lower value in the mini-PCNL group. P-values are derived from the independent-samples t-test (continuous variables; df=213) or Pearson chi-squared test (categorical variables; df=1). PCNL: percutaneous nephrolithotomy

Outcome	Mini-PCNL (n=100)	Standard PCNL (n=115)	Test statistic	P-value
Stone-free at 4 weeks, n (%)	92 (92)	108 (93.9)	χ²=0.30	0.58
Operative time (minutes), mean±SD	80.9±18.2	76.4±21.3	t=1.65	0.10
Hemoglobin decrement (g/dL), mean±SD	0.95±0.56	1.48±0.71	t=-6.01	<0.001
Hospital stay (days), mean±SD	2.4±0.8	3.1±1.1	t=-5.27	<0.001
Overall complications, n (%)	14 (14)	22 (19.1)	χ²=1.01	0.31

Stone clearance was equivalent, with both groups clearing more than nine in 10 stones. This is the finding of greatest consequence, since it alone could have justified retaining a wider tract. The two significant results were instead measures of what the operation cost the patient, and their concordance is informative: they moved in the same direction, and discharge occurred earlier despite equivalent clearance, indicating that the shorter stay reflects recovery from the access tract rather than the completeness of the operation. The weight of the hemoglobin difference depends on baseline reserve, mattering little at normal preoperative values but potentially determining whether an anemic patient crosses the transfusion threshold. The distribution of complications was similar at every Clavien-Dindo grade, the great majority minor and managed without intervention. Major complications requiring procedural intervention occurred in six patients overall, with a single grade IVa event, urosepsis requiring intensive care in a standard PCNL patient, and no deaths (Table 3).

**Table 3:** Stone clearance and postoperative complications graded by the Clavien-Dindo classification *Fisher's exact test (two-tailed) for all comparisons, owing to expected cell counts <5; p<0.05 is considered significant. No comparison reached statistical significance. Each patient is counted once, under the highest Clavien-Dindo grade sustained; itemized events therefore sum to the patient count of their grade. PCNL: percutaneous nephrolithotomy; DJ:, double-J; URS: ureteroscopy; LA: local anesthesia; SIRS: systemic inflammatory response syndrome; UTI: urinary tract infection; ICU: intensive care unit

Outcome/complication (Clavien-Dindo grade)	Mini-PCNL (n=100)	Standard PCNL (n=115)	P-value*
Efficacy			
Clinically significant residual fragments	8 (8)	7 (6.1)	0.603
Complications: overall			
Patients with ≥1 complication	14 (14)	22 (19.1)	0.362
Patients with no complication	86 (86)	93 (80.9)	-
Grade I			
Transient fever <38.5°C (antipyretics only)	4 (4)	5 (4.3)	1.000
Prolonged analgesic requirement/flank pain	2 (2)	3 (2.6)	1.000
Self-limiting hematuria (no transfusion)	1 (1)	2 (1.7)	1.000
Transient urine leak, resolved ≤48 h	1 (1)	1 (0.9)	1.000
Grade II			
UTI/fever requiring escalation of antibiotics	2 (2)	3 (2.6)	1.000
Blood transfusion	1 (1)	3 (2.6)	0.625
SIRS: IV antibiotics + fluid resuscitation (no ICU)	1 (1)	1 (0.9)	1.000
Grade IIIa			
Persistent urine leak → DJ stent under LA/sedation	1 (1)	1 (0.9)	1.000
Angio-embolization for delayed bleeding	0 (0)	1 (0.9)	1.000
Grade IIIb			
URS for steinstrasse/obstructing fragment	1 (1)	1 (0.9)	1.000
Grade IVa			
Urosepsis requiring ICU ± vasopressor support	0 (0)	1 (0.9)	1.000
Grade IVb	0 (0)	0 (0)	-
Grade V (death)	0 (0)	0 (0)	-
Minor complications (grades I-II)	12 (12)	18 (15.7)	0.555
Major complications (grades ≥III)	2 (2)	4 (3.5)	0.688

A non-significant result may reflect either a genuine absence of effect or insufficient power. Standardized effect sizes separate the three null results into two categories: stone clearance carries a negligible effect estimated with narrow confidence limits, whereas operative time and complications carry effects of clinically meaningful magnitude assessed with power far below adequacy. Both significant outcomes carry moderate to large effects with power exceeding 99% (Table 4).

**Table 4:** Effect size and achieved statistical power for each outcome CI: confidence interval; d: Cohen's d for continuous outcomes, computed as mini-PCNL minus standard PCNL and standardized to the pooled standard deviation; RR: risk ratio for categorical outcomes, expressed as mini-PCNL relative to standard PCNL. Magnitude follows conventional thresholds for Cohen's d (negligible <0.2, small 0.2-0.5, moderate 0.5-0.8, large ≥0.8). Achieved power is the post hoc power to detect the observed effect at a two-sided alpha of 0.05. PCNL: percutaneous nephrolithotomy

Outcome	Effect size (95% CI)	Magnitude	Achieved power
Stone-free at 4 weeks	RR 0.98 (0.91 to 1.06)	Negligible	8%
Operative time	d 0.23 (-0.04 to 0.49)	Small	38%
Hemoglobin decrement	d -0.82 (-1.10 to -0.54)	Large	>99%
Hospital stay	d -0.72 (-1.00 to -0.44)	Moderate	>99%
Overall complications	RR 0.73 (0.40 to 1.35)	Moderate	17%

Expressed as absolute between-group differences with confidence intervals, the same pattern is visible: the intervals for hemoglobin decrement and hospital stay lie clear of the line of no difference, whereas those for stone clearance, operative time, and complications all cross it and are wider for the categorical outcomes (Figure 2).

**Figure 2:**
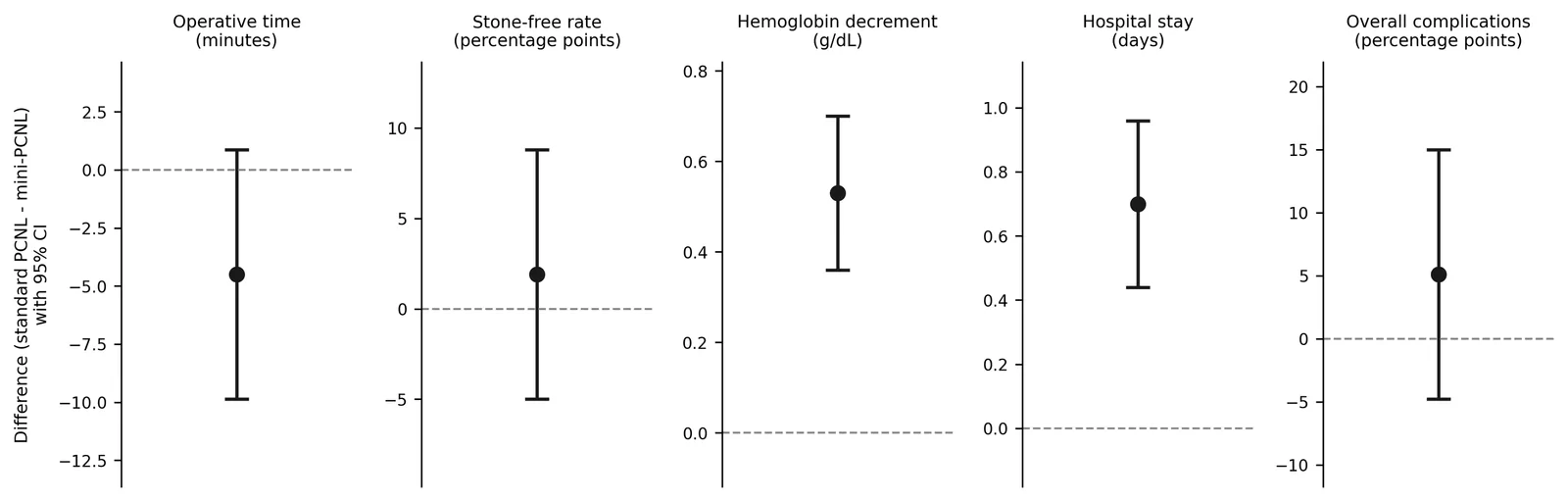
Absolute between-group differences with 95% CI for each study outcome Each panel shows the difference between groups (standard PCNL minus mini-PCNL) with its 95% CI; the dashed line marks no difference. Positive values favor mini-PCNL for all outcomes except operative time, where a negative value indicates a shorter procedure with standard PCNL. Differences are expressed in the native units of each outcome, in percentage points for stone-free rate and overall complications. PCNL: percutaneous nephrolithotomy

## Discussion

In this cohort, tract miniaturization reduced hemoglobin decrement by approximately one-third and shortened hospitalization by approximately 0.7 days, without detectable loss of stone clearance or significant prolongation of operative time. The finding is notable less for its direction than for the stone burden at which it was observed: above 2 cm, where a larger working channel might most plausibly be expected to justify its hemorrhagic cost, that justification did not materialize.

Our stone-free rates are concordant with the literature. In the pooled analysis by Qin et al. of seven randomized trials confined to stones larger than 2 cm, comprising 1,407 mini-PCNL and 1,436 standard PCNL cases, clearance was statistically identical (risk ratio 1.01; 95% CI 0.98 to 1.04), with no difference against either 30 Fr or 24 Fr comparators [6]. Equivalence extends to higher burdens: Khadgi et al., comparing 83 mini-PCNL and 70 standard PCNL procedures for staghorn calculi, reported stone-free rates of 83% and 88.6% (p=0.339) [8], while Mishra et al. found comparable clearance for 1-2 cm stones [9]. Clearance therefore appears insensitive to tract caliber from 1 cm to staghorn disease.

The mechanism is anatomical rather than technical. Blood loss during PCNL correlates with the caliber of the access sheath and the extent of parenchymal disruption at the nephrostomy tract [10]. Because puncture technique, dilatation method, lithotrite, and exit strategy were uniform across both groups, tract caliber remains the principal variable distinguishing them. The comparison is particularly close with Khadgi et al., whose technique mirrors our own and who observed a significant hemoglobin decrease with standard PCNL alongside a markedly higher transfusion rate (12.9% vs. 2.4%) [8]. Our transfusion rates were far lower in both arms and directionally similar, but with only four events in total, the study was not powered for this endpoint.

An important qualification concerns the caliber of the comparator. Pooled subgroup data indicate that the hemoglobin and transfusion advantage of mini-PCNL is demonstrable against 30 Fr but not against 24 Fr access [6], and the randomized pilot of McCahy et al. found no advantage over 24 Fr standard PCNL [11]. Because our standard arm spanned 24-30 Fr, the difference observed here likely represents a blend of these comparisons, and our effect size should not be extrapolated to units already operating predominantly at 24 Fr.

Shorter hospital stay is consistent with pooled findings and with the randomized data of Güler et al. in large stones [12], though smaller than the median six versus three days reported in staghorn disease [8], since our exclusion of staghorn and multi-tract cases removed the patients in whom prolonged recovery is concentrated. Operative time was longer with mini-PCNL, consistent with pooled data favoring standard PCNL [6]; slower fragment evacuation through a narrower nephroscope is the expected trade-off, mitigated here by hydrodynamic clearance through the sheath and likely further modifiable through suction-assisted sheaths and laser lithotripsy.

Several limitations constrain inference. Allocation followed surgeon preference rather than randomization, so confounding by indication cannot be excluded, and stone density and validated complexity scoring were not captured. Transfusion events were too few for meaningful comparison. The study was single-center and performed by surgeons experienced in both techniques, limiting generalizability to units earlier on the learning curve, and long-term recurrence and renal functional outcomes lay beyond the study window. These constraints place the findings in a confirmatory rather than definitive role.

## Conclusions

Tract caliber, rather than the percutaneous approach itself, appears to govern hemorrhagic morbidity when treating renal calculi larger than 2 cm. Miniaturized access achieved the same standard of stone clearance as standard-caliber access while producing less blood loss and permitting earlier discharge, and the modest prolongation of operative time it incurred was not clinically meaningful.

The practical implication is that stone size above 2 cm is not by itself a reason to select a wider tract. In units experienced with the technique, mini-PCNL is a reasonable default for non-staghorn calculi of this burden rather than a selective alternative reserved for smaller stones. Standard-caliber access retains a clear role where stone burden is very high, where morphology is complex or staghorn, and where operative efficiency outweighs the value of reduced blood loss.

These conclusions rest on non-randomized data from a single center and support rather than establish this position. A randomized comparison against a uniform-caliber standard arm, powered for transfusion as the primary hemorrhagic endpoint, remains the necessary next step.
